# Preservation of pelvic floor muscles contributes to early continence recovery after robot-assisted radical prostatectomy

**DOI:** 10.1371/journal.pone.0275792

**Published:** 2022-10-07

**Authors:** Masaki Nakamura, Yuta Yamada, Yusuke Sato, Kazuki Honda, Daisuke Yamada, Taketo Kawai, Yoshiyuki Akiyama, Motofumi Suzuki, Haruki Kume

**Affiliations:** 1 Department of Urology, Graduate School of Medicine, The University of Tokyo, Tokyo, Japan; 2 Department of Urology, NTT Medical Center Tokyo, Tokyo, Japan; University of Alberta, CANADA

## Abstract

**Purpose:**

Postoperative recovery of urinary continence has a great impact on quality of life for patients undergoing robot-assisted radical prostatectomy (RARP). A variety of surgical techniques including reconstruction of the periurethral structure have been introduced, and yet there are no effective methods that promote early urinary continence recovery after surgery. We hypothesized that the preservation of pelvic floor muscle structure could be responsible for early recovery of urinary continence after surgery.

**Methods:**

A total of 94 consecutive patients who underwent RARP at our hospital were enrolled in this study. Operative video records were reviewed and the severity of pelvic floor muscle injury was classified according to the scoring system that we devised in this study. Briefly, damage of pelvic floor muscles was classified into 4 categories; intact, fascial injury, unilateral muscle injury, and bilateral muscle injury. The volume of urinary incontinence was measured for 2 days after removal of the urethral catheter, and the incontinence ratio (amount of incontinence/total volume of urine per day) was calculated. Predictive factors for immediate incontinence after catheter removal were identified by multivariate regression analysis.

**Results:**

The severity of puboperineal muscle injury was significantly associated with the early incontinence ratio after catheter removal (*p* < 0.001). Age at surgery and severity of puboperineal muscle injury were independent predictors for early incontinence after catheter removal.

**Conclusion:**

Preservation of the pelvic floor muscle, particularly the puboperineal muscle is an important factor for early continence recovery after RARP.

## Introduction

Robot-assisted radical prostatectomy (RARP) has become a standard procedure as a surgical treatment for organ-confined prostate cancer. Although the incontinence rate following RARP is lower than that with open radical prostatectomy, early urinary continence recovery after RARP is a still debatable issue.

We previously reported that age, prostate volume, and pT stage are potential predictors of early continence recovery after RARP [1]. The incontinence rate at 2 weeks after RARP was 89.9% for patients under 67-years old, and 96.3% for those older than 67years, respectively. Incontinence rates after RARP vary between 4% and 31% with no pad definition, and between 8% to 11% with no or safety pad definition [2].

Rocco’s stitch is one of the most popular technique for posterior reconstruction during RARP [3]. In addition to this technique, several effective methods for periurethral reconstruction have been reported [4–6]. However, some of these techniques seem to damage the anatomical structures rather than preserving them.

We hypothesized that the preservation of pelvic floor muscle structure could be attributable to early continence recovery after RARP, and thereby retrospectively reviewed operative records of consecutive 94 patients who underwent the surgery in our hospital.

## Materials and methods

### Patients

Ninety-four consecutive patients diagnosed with localized prostate cancer who underwent RARP between January and November in 2019 were enrolled. Urethral catheters were removed on postoperative day 6. This study was approved by the ethics committee at the university of Tokyo hospital and was conducted in accordance with the Helsinki Declaration. During RARP, we have performed Rocco’s stitch for posterior reconstruction. Bladder necks were preserved and bidirectional running vesicourethral anastomosis were performed with double ended 3–0 MONOCRYL^®^ in all the cases. 16 Fr urethral catheters were placed until removal on postoperative day 6.

### Classification of pelvic floor muscle injury

The puboperinealis muscle originates from the body of the pubis and courses posteriorly to attach the dorsal medium raphe. The pubococcygeus muscle originates from the body of the pubis and courses posteriorly to attach along the midline as far back as the coccyx [7].

**Fig 1:**
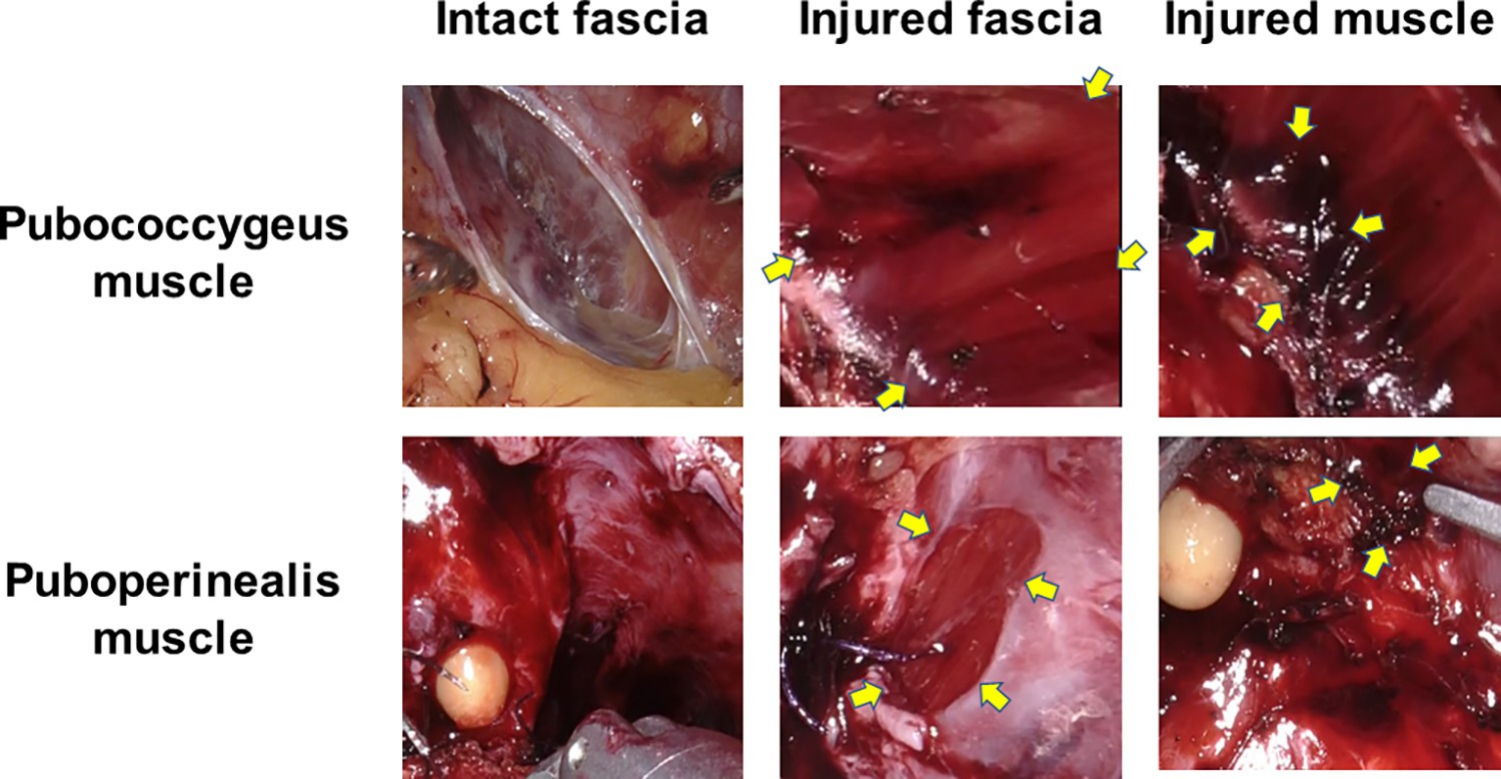
Representative pictures of intact fascia, injured fascia, and injured muscle of the pubococcygeus and puboperineal muscles. Yellow arrows indicate the area of injury.

We defined pelvic floor muscle injury according to 4 categories; i.e., 0: intact, 1: muscular fascia injury; only the fascia of pubococcygeal muscle or puboperineal muscle is peeled off, 2: unilateral muscle fiber injury; unilateral substantial damage, loss, or heat damage of the muscle fiber, 3: bilateral muscle fiber injury; bilateral substantial damage, loss, or heat damage of the muscle fiber (Fig 1). Two surgeons (M.N. and Y.Y.) shared the category of fascial and muscular damage before review and scored the damage of the puboperineal muscle and pubococcygeal muscle independently. When the evaluation of two surgeons did not match, they discussed and unified the evaluation.

### Calculation of incontinence ratio

The amount of urinary incontinence was calculated as “weight of the wet pad” subtracted by “the weight of the clean pad”. Incontinence ratio was calculated as the amount of urinary incontinence divided by the total volume of urine per day.

### Statistical analysis

Kruskal–Wallis test was applied to compare the incontinence ratio between different severities of pelvic floor muscle injury. Post-hoc Mann Whitney U test with Bonferroni correction were applied accordingly. Spearman’s test was used to examine the correlation between incontinence ratio and age. Pearson’s correlation coefficient was calculated to show the correlation between nerve-paring and severity of puboperineal muscle injuries. Multivariate regression analysis was performed to identify predictor of early urinary continence recovery. P value of 0.008 for MW with B correction, 0.05 for others were considered statistically significant.

## Results

**Fig 2:**
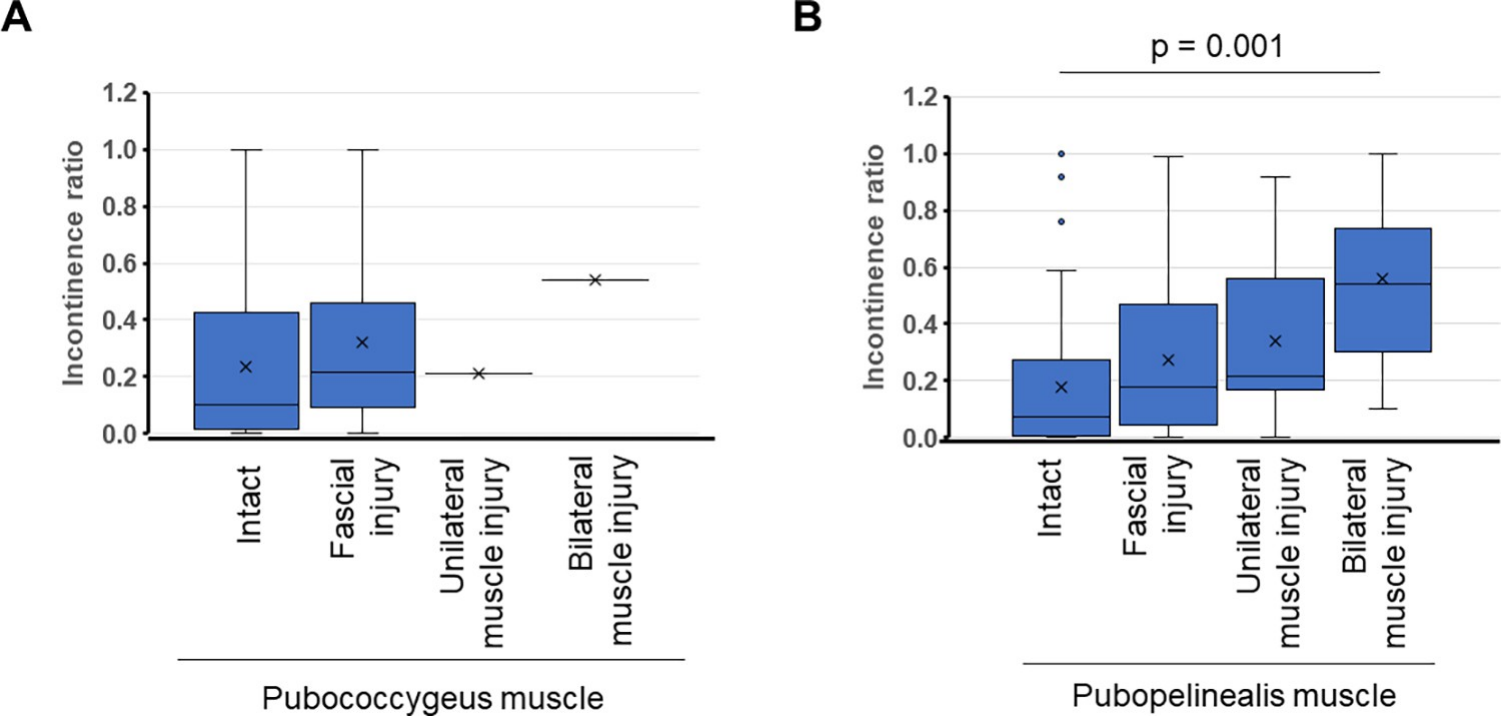
A) Boxplot of incontinence ratio 2 days after urethral catheter removal. X axis: magnitude of pubococcygeus muscle injury. B) Boxplot of incontinence ratio two days after urethral catheter removal. X axis: magnitude of puboperineal muscle injury.

**Fig 3:**
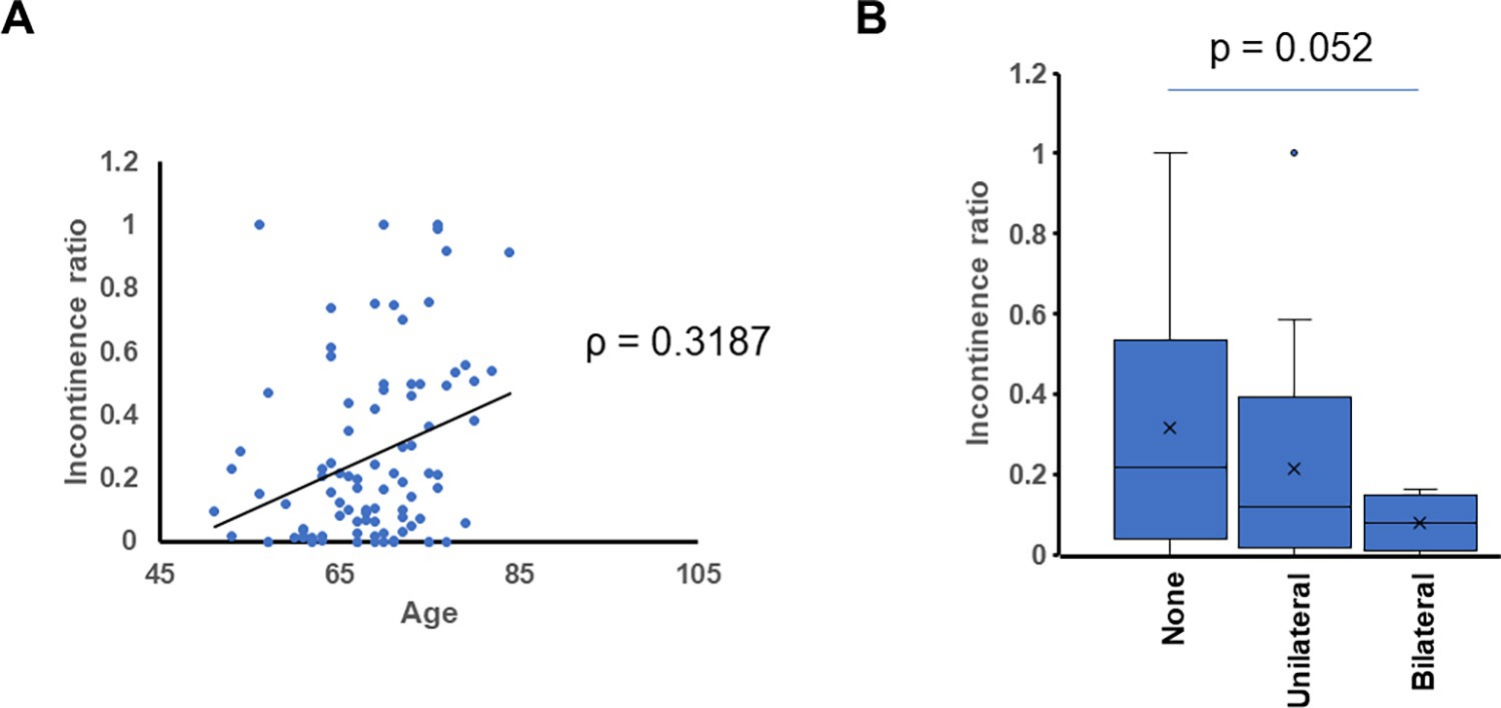
A) Dot plot showing the relationship between age and incontinence ratio. Spearman’s ρ = 0.3187, p = 0.002. B) Box plot of incontinence ratio at 2 days after urethral catheter removal. X axis: status of nerve sparing.

Patient demographics are shown in Table 1. The median age of the patients was 69, and the median PSA value was 6.74 ng/mL The average of daily urine volume at 2 days after urethral catheter removal for category 0, 1, 2, and 3 were 1937 mL, 2326 mL, 2004 mL, and 1860 mL, respectively. There was no statistically significant difference between groups. No patient showed urethral stricture within 2 days after urethral catheter removal. The severity of puboperineal muscle injury was positively associated with the incontinence ratio at 2 days after urethral catheter removal (p < 0.05). We have detected two cases of pubococcygeus muscle injury. The number of occurences was too small to draw a conclusion about the relationship between the severity of pubococcygeus muscle injury and urinary incontinence (Fig 2A and 2B). There was a tendency for age to positively correlated with the incontinence ratio at 2 days after urethral catheter removal (Spearman ρ = 0.3187; *p* = 0.002) (Fig 3A).

**Table 1:** Patient demographics.

No. patients	94
Median age (range) (y)	69 (51―84)
Median BMI (range)	23.9 (16.9―31.1)
Median PSA (range) (ng/mL)	6.74 (1.07―76.0)
Median console time (range) (min)	136.5 (79―435)
Median estimated blood loss (range) (mL)	200 (0―2150)
Median prostatic volume (range) (cm^3^)	30.3 (14.3―74.0)
Nerve sparing	
Not performed	55
Unilateral	29
Bilateral	10
No. pubococcygeus muscle injury	
Intact	69
Fascial injury	23
Unilateral muscle injury	1
Bilateral muscle injuries	1
No. puboperineal muscle injury	
Intact	56
Fascial injury	16
Unilateral muscle injury	11
Bilateral muscle injuries	11
Comorbidity, N	
Hypertension	22
Diabetes mellitus	13
Dyslipidemia	12

Nerve-sparing surgery had a trend of having negative correlation with the incontinence ratio at 2 days after urethral catheter removal (p = 0.052), which further support our hypothesis that preserving muscle structure and nerves could simply support early recovery of the urinary continence after RARP (Fig 3B). The number of puboperineal muscle injuries in each group of nerve-sparing technique were shown in Table 2. On the same side of nerve-sparing, only 1 muscular damage was observed. There was a negative correlation between nerve-sparing and the number of puboperineal muscle injuries (Pearson’s correlation coefficient -0.296, p = 0.004).

**Table 2:** Number of patients with puboperineal muscle injury.

		Puboperineal muscle injury			
		None	Fascial injury	Unilateral muscle injury	Bilateral muscle injury
Nerve sparing	Not performed	28	8	9	10
	Unilateral	20	6	2	1
	Bilateral	8	2	0	0

Results of univariate and multivariate analysis are shown in Table 3. Multivariate regression analysis revealed that the severity of puboperineal muscle injury and age were independent predictors of urinary incontinence at 2 days after urethral catheter removal (*p* = 0.001 and 0.028, respectively).

**Table 3:** Predictors of incontinence after removal of the urethral catheter. Results of univariate and multivariate analysis.

	Univariate analysis			Multivariate analysis		
	Unstandardized regression coefficient B (95% CI)	Standardized regression coefficient β	*p-*value	Unstandardized regression coefficient B (95% CI)	Standardized regression coefficient β	*p-*value
Age^†^	0.014 (0.006, 0.022)	0.326	0.001	0.011	0.365	0.011*
BMI	0.001 (-0.023, 0.026)	0.012	0.915			
Prostate volume^†^	-0.004 (-0.009, 0.000)	-0.19	0.061	-0.003	-0.120	0.205
Nerve sparing^†^	-0.122 (-0.204, -0.039)	-0.287	0.004	-0.026	-0.571	0.569
eBlood loss	-0.00005 (0.00, 0.00)	0.060	0.560			
Pubococcygeus muscle injury	0.075 (-0.041, 0.191)	0.136	0.203			
Puboperineal muscle injury^†^	0.122 (0.114, 0.256)	0.432	<0.001	0.103	0.365	<0.001*

BMI body mass index, eBlood loss estimated blood loss, CI confidence interval

^†^ These factors were put in the multivariate regression analysis

* p-value < 0.05 was considered statistically significant.

## Discussion

The urethra is surrounded by several layers of periurethral muscular structures. From the inner layers are the urethral striated sphincter, the urethral smooth sphincter (circular fibers), and the urethral smooth sphincter (longitudinal fibers). The puboperineal muscle, the puborectal muscle, and the pubococcygeus muscle also support and surround the urethra [7].

Preservation of the nerves including the pudendal and pelvic nerves also contribute to urinary continence after RARP. Nerves innervating the pelvic floor muscles and the periurethral muscles include the pudendal nerves and the pelvic nerves [8]. Previously, the pudendal nerves have been considered to innervate voluntary outer urethral sphincter muscle, and pelvic splanchnic nerves innervate involuntary bladder neck sphincter muscles, exclusively [9]. Recently, however, the pelvic splanchnic nerves and the pudendal nerves were reported to fuse to innervate the outer urethral sphincter muscles and the pelvic floor muscles [10]. These fused nerves are reported to run along the pubococcygeus muscle and the puborectal muscle to penetrate the levator ani muscle to innervate the outer urethral sphincter muscles [10]. Therefore, damaging the pelvic floor muscles poses a risk of injuring these nerves.

Several maneuvers to prevent urinary incontinence have been reported to be effective. Among them, standard posterior reconstruction according to Rocco et al. is the most popular method for posterior reconstruction in RARP procedure [3, 11]. In addition to this technique, Porpiglia et al. reported that total anatomical reconstruction during RARP where all peri-urethral layers (i.e., median raphe to retrotrigonal layer, rabdosphincter to bladder neck sphincter, and parietal layer of pelvic fascia to visceral layer of pelvic fascia) are sutured layer to layer. The continence rates (pad free or safety pad) were 77.8%, 89.3%, 94.4%, and 98% for 1 week, 4 weeks, 12 weeks, and 24 weeks after RARP, respectively [5]. Moro et al. reported on the Complete Reconstruction of Posterior Urethral Support (COROUS) technique, where both sides of puboperineal muscles are tied together posterior to the urethra. The continence rates at 30 days after RARP were 83% and 61% for the CORPUS group and standard Rocco group, respectively [4]. Advanced Reconstruction of Vesicourethral Support (ARVUS) reported by Student et al. is another eye-catching technique where bilateral levator ani muscles are tied together with Denonviller’s fascia posterior to the urethra. The continence rate (pad free or safety pad) was 62.5%, 68.8%, and 75% at 4 weeks, 8 weeks, and 6 months after RARP, respectively [6]. As represented by the CORPUS and ARVUS technique, some methods of reconstruction utilize pelvic floor muscle structure to artificially form supportive lining for the urethral muscle. Tying bilateral muscle fascia, for example, impedes the original motion of the pelvic floor muscles.

Herein, we report that a surgical technique of preserving pelvic floor muscles and nerves with the RARP procedure contributes to immediate recovery of urinary continence after surgery. Multivariate analysis revealed that the severity of muscular injury around the urethra is a predictor of the early incontinence ratio after RARP. Preserving the innate muscular structure is necessary for early recovery of urinary continence after RARP. In this study, nerve-sparing technique negatively correlated with the number of puboperineal muscle injuries. The favorable trend between nerve-sparing and postoperative urinary continence could partly be explained by the preservation of puboperineal muscles.

Some limitations of this study should be mentioned. First, we enrolled a relatively small number of patients. Second, the study was retrospectively designed. A prospective study with a larger number of patients is required to further clarify the effect of pelvic floor muscle preservation on early recovery of urinary continence.

## Conclusion

Preservation of pelvic floor muscular structure contributes to early urinary continence recovery after RARP.

## Supporting information

S1 Data (XLSX)
